# Targeting biomolecular condensates to inhibit breast cancer

**DOI:** 10.1002/ctm2.70296

**Published:** 2025-04-01

**Authors:** Xiaoxue Zhou, Linghui Zeng, Fangfang Zhou

**Affiliations:** ^1^ School of Medicine Hangzhou City University Hangzhou China; ^2^ MOE Key Laboratory of Macromolecular Synthesis and Functionalization, Department of Polymer Science and Engineering Zhejiang University Hangzhou China; ^3^ The First Affiliated Hospital, the Institutes of Biology and Medical Sciences, Suzhou Medical College Soochow University Suzhou China

## 1 INTRODUCTION AND CHALLENGES IN BREAST CANCER

Breast cancer remains one of the most prevalent and deadly cancers worldwide, with metastasis being the primary cause of mortality.^1^ Despite significant advances in treatment, the development of resistance to conventional therapies and the lack of effective strategies to prevent metastasis continue to pose major challenges.^2^ One of the most exciting developments in cancer biology in recent years has been the discovery of liquid–liquid phase separation (LLPS), a process by which proteins and other biomolecules form membraneless organelles or condensates within cells.^3^ These condensates compartmentalise biochemical reactions, allowing for the regulation of complex cellular processes, including transcription, DNA repair, and signal transduction.^4^, ^5^, ^6^ In the context of cancer, LLPS has been implicated in the dysregulation of key oncogenic pathways.^7^, ^8^ The aberrant formation and regulation of phase‐separated condensates are increasingly recognised as critical factors driving tumourigenesis and metastasis.^9^ However, targeting these condensates for therapeutic intervention remains a significant challenge.

## 2 LLPS AND FOXM1 CONDENSATES

**FIGURE 1:**
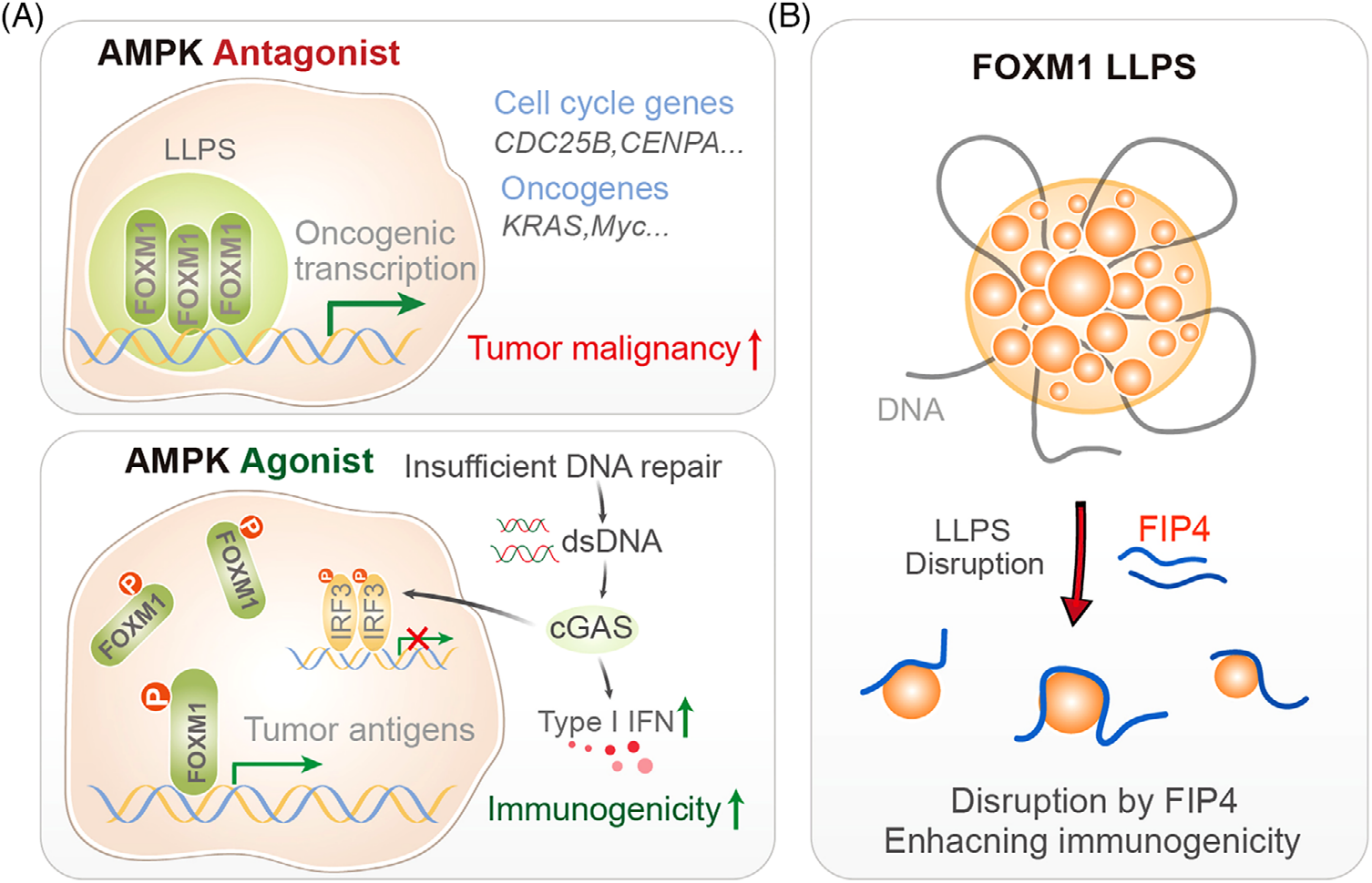
Schematic illustration of FOXM1 condensate regulation and FIP4‐mediated therapeutic effects. (A) Phase‐separated FOXM1 condensate to super‐enhancer of target genes that mediates efficient metastatic outgrowth and DNA damage repair in tumour cells, resulting in markedly enhanced malignancy; phosphorylation at Ser376 disrupts FOXM1 condensate formation, reducing DNA damage repair and metastatic gene expression. This leads to dsDNA accumulation, activation of IRF3–IFN‐I signalling and increased tumour antigen expression, enhancing tumour immunogenicity. (B) FIP4 disrupts FOXM1 condensate formation, inhibits metastatic progression and enhances tumour immunotherapy. FIP4 specifically targets FOXM1–IDR1, blocking IDR1‐mediated FOXM1 phase separation, thereby reducing oncogenic transcriptional activity and enhancing immunogenicity.

In this context, the recent discovery of the role of FOXM1 (Forkhead box protein M1) in breast cancer progression, and the development of a novel therapeutic strategy to target its phase‐separated condensates, represents a groundbreaking advancement with profound implications for clinicians and clinical researchers.^10^ In our recent study, we identified FOXM1 as a protein that undergoes LLPS in breast cancer cells.^10^ FOXM1 condensates form in the nucleus, where they compartmentalise the transcription machinery, maintaining chromatin accessibility and super‐enhancer landscapes that are crucial for tumour growth and metastasis. FOXM1 is a transcription factor that plays a critical role in cell cycle progression, DNA repair, and cellular differentiation. It is frequently overexpressed in various cancers, including breast cancer, where it drives tumour growth, metastasis, and resistance to therapy. This phase‐separated state of FOXM1 enhances its transcriptional activity, driving the expression of oncogenic genes that promote tumour progression (Figure 1A).

## 3 AMP‐ACTIVATED PROTEIN KINASE‐MEDIATED DISRUPTION OF FOXM1 CONDENSATES

A key finding of our research is that the formation of FOXM1 condensates can be disrupted by the activation of AMP‐activated protein kinase (AMPK), a cellular energy sensor that plays a critical role in maintaining metabolic homeostasis. We discovered that AMPK phosphorylates FOXM1 at a specific site Ser376 within its intrinsically disordered region (IDR), leading to the dissolution of FOXM1 condensates. This phosphorylation event introduces electrostatic repulsion, preventing FOXM1 from undergoing LLPS and aggregating into functional condensates. The disruption of FOXM1 condensates by AMPK activation has several important consequences. First, it reduces the transcriptional activity of FOXM1, leading to the downregulation of oncogenic genes that drive tumour growth and metastasis. Second, it results in the accumulation of double‐stranded DNA (dsDNA) in the cytoplasm, which activates the cGAS–STING pathway, a key component of the innate immune response. This activation enhances tumour immunogenicity, making the cancer cells more susceptible to immune‐mediated destruction (Figure 1A).

## 4 DEVELOPMENT OF FIP4‐A NOVEL THERAPEUTIC PEPTIDE

Building on these findings, we developed a peptide‐based therapeutic strategy to target FOXM1 condensates. The peptide, named FIP4 (FOXM1‐interfering peptide 4), mimics the phosphorylated form of FOXM1 and binds to its IDR, preventing the formation of condensates. FIP4 effectively disrupts FOXM1 LLPS, reducing its transcriptional activity and inhibiting tumour growth and metastasis in preclinical models (Figure 1B). In animal studies, FIP4 demonstrated significant anti‐tumour effects, reducing tumour volume and metastatic burden in breast cancer models. Importantly, FIP4 also enhanced tumour immunogenicity, suggesting that it could be used in combination with immunotherapies to improve treatment outcomes. The development of FIP4 represents a significant step forward in the quest to target transcription factors like FOXM1, which have traditionally been considered “undruggable.”

## 5 CLINICAL IMPLICATIONS AND FUTURE DIRECTIONS

The implications of our findings for clinical practice are profound. First, the identification of FOXM1 condensates as a key driver of breast cancer progression provides a new therapeutic target. By disrupting these condensates, we can potentially halt tumour growth and prevent metastasis, addressing a major unmet need in breast cancer treatment. Second, the role of AMPK in regulating FOXM1 condensates suggests that AMPK activators, such as metformin, could be repurposed as anti‐cancer agents. Metformin, a widely used diabetes drug, has already shown promise in preclinical and clinical studies as an anti‐cancer agent. Our findings provide a mechanistic basis for its anti‐tumour effects and suggest that it could be used in combination with other therapies to enhance its efficacy. Third, the development of FIP4 opens up new possibilities for peptide‐based cancer therapies. Peptides offer several advantages over small molecules, including high specificity and low toxicity. FIP4, in particular, has shown excellent tumour‐targeting capabilities and could be further developed for clinical use. Its ability to enhance tumour immunogenicity also makes it an attractive candidate for combination therapies with immune checkpoint inhibitors.

While our findings are promising, several challenges remain. First, the safety and efficacy of FIP4 need to be rigorously tested in clinical trials. Second, the potential for resistance to FIP4 and other FOXM1‐targeting therapies needs to be explored. Finally, the broader applicability of targeting LLPS in other cancers and diseases warrants further investigation.

In conclusion, our work on FOXM1 condensates represents a significant advance in our understanding of breast cancer biology and provides a novel therapeutic strategy with the potential to transform patient care. By targeting the phase‐separated state of FOXM1, we can disrupt key oncogenic pathways, enhance tumour immunogenicity, and ultimately improve outcomes for patients with breast cancer. This research underscores the importance of exploring new biological paradigms, such as LLPS, in the quest to develop more effective cancer therapies. For clinicians and clinical researchers, these findings offer new hope and new tools in the fight against breast cancer. As we continue to unravel the complexities of cancer biology, the potential for innovative therapies like FIP4 to make a real difference in patients' lives becomes increasingly clear.

## AUTHOR CONTRIBUTIONS

Xiaoxue Zhou, Linghui Zeng and Fangfang Zhou conceptualised and wrote the article.

## CONFLICT OF INTEREST STATEMENT

The authors declare no conflict of interest.

## ETHICS STATEMENT

The authors have nothing to report.

## Data Availability

Data sharing not applicable to this article as no datasets were generated or analysed during the current study.
